# Prognostic Role of the Pretreatment C-Reactive Protein/Albumin Ratio in Solid Cancers: A Meta-Analysis

**DOI:** 10.1038/srep41298

**Published:** 2017-01-27

**Authors:** Nan Li, Guang-Wei Tian, Ying Wang, Hui Zhang, Zi-hui Wang, Guang Li

**Affiliations:** 1 Department of Radiation Oncology, The First Affiliated Hospital of China Medical University, Shenyang 110001, China; 2 Laboratory of Gastrointestinal Onco-Pathology, Cancer Institute & General Surgery Institute, The First Hospital of China Medical University, Shenyang 110001, China; 3 Department of Gastroenterology, Beijing Tian Tan Hospital, Capital Medical University, Beijing 100050, China; 4 Department of Biomedical Science, Ohio University, Irvine Hall, Athens, Ohio 45701, USA

## Abstract

The C-reactive protein/albumin ratio (CAR) has been shown to play a significant prognostic role in several cancers. We aimed to comprehensively explore the potential role of the CAR as a prognostic indicator in solid cancers. In this meta-analysis, we collected data from 10 studies that examined the association between serum CAR and overall survival in patients with cancer. This meta-analysis included 4592 tumor patients. The eligible studies were found through the PubMed and Web of Science databases updated on 6 Oct 2016. The pooled hazard ratio (2.01, 95% CI: 1.58–2.56, p < 0.001) indicated that high CAR yielded worse survival in different cancers. Subgroup analyses showed a significant association between CAR and prognosis, regardless of the cutoff value, cutoff value selection, treatment method, country, sample size, stage and cancer type. This meta-analysis suggests that CAR may be a potential prognostic marker in solid cancers. However, further large prospective studies should be conducted to explore the critical role of CAR in survival of cancer patients.

Due to increasing morbidity and mortality, cancer remains a global and growing, but not uniform, problem^1^. Despite decades of research, relatively few biomarkers are routinely used in clinics for specific types of cancer (e.g., CA-125^2^ and PSA^3^ in ovarian and prostate cancers, respectively). Most patients still have either regional or distant metastatic disease when diagnosed, which always means a complicated therapy and poor prognosis^4^. There is a demand for reliable and clinically applicable pan-cancer biomarkers to obtain additional prognostic information.

Approximately a quarter of cancer patients show correlations with inflammation, and previous studies have claimed that inflammation is a major hallmark of cancer^5^,^6^. Several studies have supported the hypothesis that inflammation is closely related to tumor development, progression, and metastatic dissemination, as well as resistance to treatment^7^. Compared to one of these traditionally recognized methods, prostate-specific antigen testing for prostate cancer^3^, which assesses systemic inflammation conditions within the tumor by a peripheral blood test during diagnosis or before treatment, is a relatively cheap and convenient method. Fortunately, recent studies have demonstrated that various inflammatory biomarkers, such as the neutrophil-lymphocyte ratio (NLR) and the platelet-lymphocyte ratio (PLR), play significant roles in various cancers^8^,^9^,^10^. C-reactive protein is a representative and routinely measured inflammatory marker, and elevated levels have been associated with treatment outcomes in different malignancies^11^,^12^. Furthermore, the Glasgow Prognostic Score (GPS) and the modified Glasgow Prognostic Score (mGPS), which are determined based on the serum levels of C-reactive protein and albumin, have been linked to outcomes of cancer patients^13^,^14^. The C-reactive protein to albumin ratio (CAR), a novel inflammation-based prognostic score, is also based on these two factors^15^. Recently, several studies have revealed that the CAR may be a pan-cancer prognostic marker in many types of cancers, including hepatocellular carcinoma^16^ and esophageal squamous cell carcinoma^17^.

Although there is a relationship between high CAR and human cancer, most studies reported thus far have had restricted sample sizes or discrete outcomes. Here, we performed a meta-analysis of data from published studies to comprehensively and quantitatively evaluate its prognostic value in various cancers.

## Materials and Methods

This meta-analysis was conducted following the Preferred Reporting Items for Systematic Reviews and Meta-analysis (PRISMA) criteria^18^.

### Literature search and study selection

Two investigators independently searched for eligible studies in PubMed and Web of Science to evaluate the prognostic value of CAR in patients with cancer. Based on the search strategy, which included the following search terms: (“C-reactive protein Albumin ratio” or “C-reactive protein to Albumin ratio” or “C-reactive protein/Albumin ratio” or “CAR”) and (“cancer” or “carcinoma”) and (“prognosis” or “survival”), we identified these studies until Oct 6, 2016.

### Inclusion and exclusion criteria

Studies with the following criteria were included in the meta-analysis: (1) patients with any type of solid cancers were studied; (2) the prognostic value of the pretreatment CAR was evaluated; (3) hazard ratio (HR) for overall survival (OS) was evaluated with multivariate analysis using the Cox proportional hazard model; (4) a definite cutoff value of CAR was given; (5) publications were full-text studies in English. The exclusion criteria were as follows: (1) hematological malignances; (2) letters, reviews, case report or laboratory studies; (3) insufficient information for data extraction; (4) studies had duplicate data or repeat analysis.

### Data extraction and quality assessment

The data from all eligible studies were independently reviewed and extracted by two investigators. Each disagreement was assessed until the investigators reached a consensus to guarantee the accuracy of the information extracted. The extracted data from every study included the first author, year of publication, country of origin, total number of cases, cancer type, study type, cut-off value, cut-off selection methods, range of CAR, treatment strategy, stage, follow-ups, age and HRs for OS and disease-free survival (DFS), as well as their 95% confidence intervals (CIs) and p values for the correlation between CAR and prognosis.

The qualities of the included studies were assessed using the Newcastle–Ottawa Quality Assessment Scale (NOS)^19^. The NOS comprised three parameters of quality: selection (0–4 points), comparability (0–2 points), and outcome assessment (0–3 points). The maximum score is 9 points, and NOS scores of ≥7 were defined as high-quality studies^20^. Any disagreement was resolved by discussion.

### Statistical analysis

This meta-analysis was performed with STATA version 12.0 (Stata Corp LP, TX, USA) and RevMan software (version 5.3; The Cochrane Collaboration). HRs and their 95% CIs were extracted from each study to calculate pooled HRs. When they were not reported directly in the original study, we estimated the HR through the extracted data from the Kaplan-Meier curve using the methods published by Tierney *et al*.^21^. The heterogeneity of the pooled results was measured using Cochran’s Q test and Higgins I-squared statistic. Significant heterogeneity was defined as p < 0.1 or I^2^ > 50%. The random-effects model (DerSimonian-Laird method)^22^ was used to analyze the pooled HRs when heterogeneity was significant; otherwise, the fixed-effects model (Mantel Haenszel method)^23^ was applied. Publication bias was formally investigated by three methods, the Begg’s^24^ and Egger’s tests^25^, and the “trim and fill” method^26^. The trim-and-fill method estimates the number of missing studies needed. Subgroup analysis was performed on the basis of cutoff value, cutoff value selection, treatment method, country, sample size, stage and cancer type. The differences between the subgroups were assessed using RevMan software. Sensitivity analysis was used to examine the stability of the pooled results using STATA software. Furthermore, linear regression analysis was performed to evaluate the correlation of the CAR cutoff value and log (CAR cutoff value) with the HR for OS using GraphPad Prism Software 5 (GraphPad Software Inc., San Diego, CA, USA).

## Results

**Figure 1:**
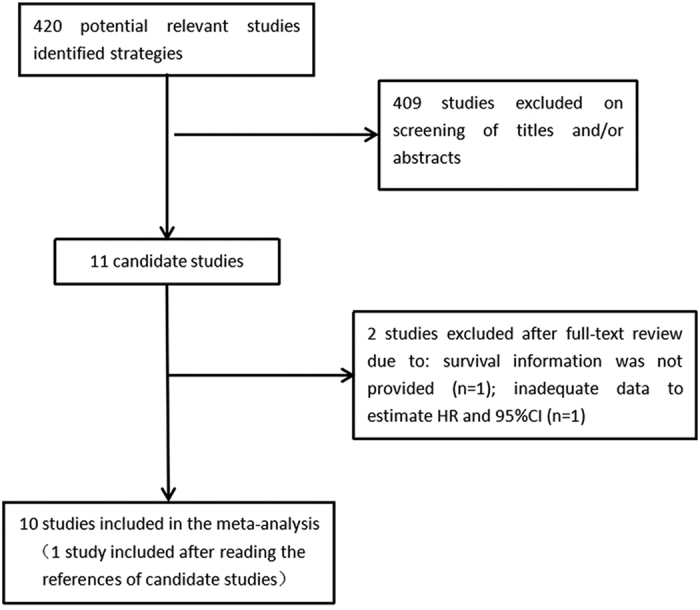
The flow chart of literature selection.

The selection process is shown in Fig. 1. We identified 420 relevant studies from the first search strategy. After screening the titles and abstracts, 11 potential studies were selected. Among these studies, Masatsune Shibutani *et al*.^27^ evaluated the prognostic significance of CAR with relapse-free survival (RFS) and cancer-specific survival (CSF) instead of OS, while one study failed to obtain a definite HR because the tumor patients in this study were classified into three groups based on two different CAR cutoff values^28^. After reading these studies, we found another publication which evaluated the prognostic value of CAR in patients with small-cell lung cancer (SCLC)^29^. Finally, 10 eligible studies were selected that met the inclusion criteria in this meta-analysis.

The major characteristics of this meta-analysis are shown in Table 1. These 10 retrospective studies compromised 4592 patients with hepatocellular carcinoma (HCC)^16^, esophageal squamous cell carcinoma (ESCC)^17^,^30^, gastric cancer (GC)^31^, small-cell lung cancer (SCLC)^29^, colorectal cancer (CRC)^32^,^33^, pancreatic cancer^34^,^35^ and nasopharyngeal carcinoma (NPC)^36^. All studies were published between 2015 and 2016 and were from China (n = 7) or Japan (n = 3). Based on different treatment methods, the studies were divided into three groups, including with-surgery (n = 6) and no-surgery (n = 4) treatment. Only two study presented the HR for both DFS and OS, and the HRs and their 95% CIs for OS were directly extracted from the rest of the studies. All studies conducted a multivariable analysis of OS. The quality of 10 studies was evaluated using the Newcastle–Ottawa Quality Assessment Scale (NOS) (Table 2).

**Figure 2:**
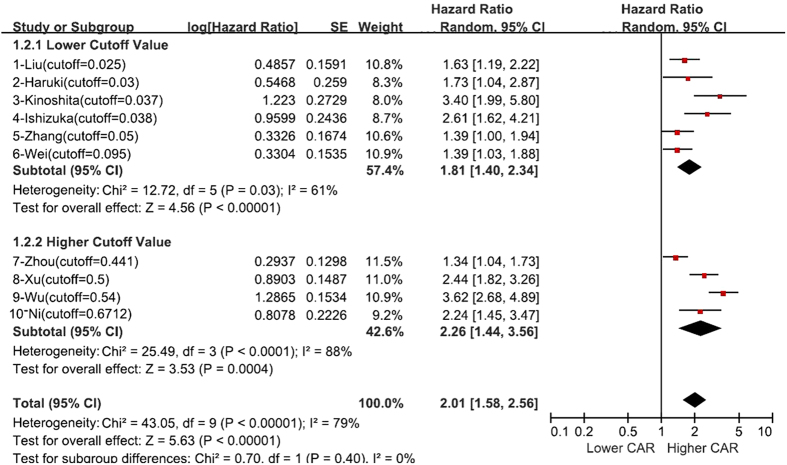
Forest plot of hazard ratio (HR) for the association of CAR with overall survival (OS) and subgroup analysis by cutoff value.

The pooled results showed that patients with a high pretreatment CAR had significantly poorer OS than those with low CAR (HR: 2.01, 95% CI: 1.58–2.56, I^2^ = 79%, p < 0.001, Fig. 2). For further exploration of the heterogeneity, subgroup analyses were conducted.

**Figure 3:**
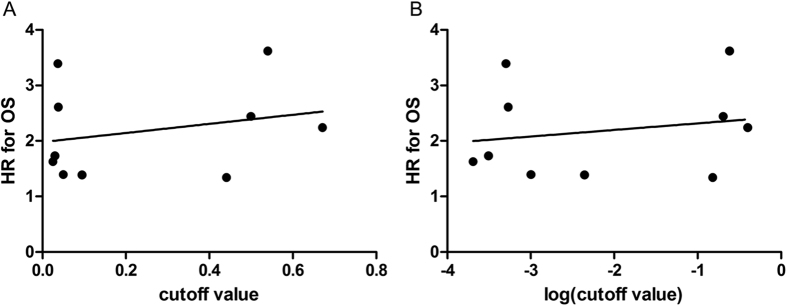
The correlation of cutoff value and log (cutoff value) of the HR for OS using linear regression analysis.

We performed subgroup analysis of OS based on cutoff value because there was a large range of change between each study. First, we evaluated the correlation of cutoff value and HR for OS using linear regression analysis. The results showed that there was no association between cutoff value and HR for OS (r^2^ = 0.0658, p = 0.474) (Fig. 3A). Because we did not know whether the CAR in each study was normally distributed, the correlation of log (cutoff value) and HR for OS was analyzed. Moreover, there was no association between log (cutoff value) and HR for OS (r^2^ = 0.0377, p = 0.591) (Fig. 3B). We classified the cutoff values into the lower cutoff group, which had a cutoff value lower than 0.1, and the higher cutoff group, where the cutoff values ranged from 0.4 to 0.7. A combined analysis showed that a higher CAR, which was higher than that of cutoff, was associated with poor OS both in the lower cutoff group (HR: 1.81, 95% CI: 1.40–2.34, p < 0.001) and the higher cutoff group (HR: 2.26,95% CI: 1.44–3.56, p = 0.004). There was no statistically significant difference between these groups (p for subgroup difference = 0.40) (Fig. 2). In all included studies, 7 studies reported that the cutoff value was selected by the receiver operating characteristic (ROC), and 3 studies selected the cutoff value based on Cutoff Finder, which was a web-based system, R software-engineered, designed by Budczies J *et al*.^37^. In cutoff selection, subgroup analysis showed that elevated CAR was positively related to poor HR both in the ROC group (HR: 2.05, 95% CI: 1.64–2.57, p < 0.001) and the Cutoff Finder group (HR: 1.89, 95% CI: 1.01–3.51, p = 0.046) (p for subgroup difference = 0.80). When different treatment methods were considered, elevated CAR was positively related to poor OS both in the with-surgery group (HR: 2.03, 95% CI: 1.56–2.64, p < 0.001) and the no-surgery group (HR: 1.97 95% CI: 1.20–3.23, p = 0.008) (p for subgroup difference = 0.92). In the subgroup analyses by country, we found increased CAR predicted a worse OS for Chinese (HR: 1.87, 95% CI: 1.40–2.50, p < 0.001) and Japanese (HR: 2.47, 95% CI: 1.70–3.59, p < 0.001) patients (p for subgroup difference = 0.25). After stratification by sample size, the pooled HRs were 1.69 (95% CI: 1.35–2.12) for studies with more than 300 cases and 2.69 (95% CI: 1.90–3.81) for studies with less than 300 cases (p for subgroup difference = 0.03). When different stages were considered, the hazard ratios for the effect of CAR on OS were 2.00 (95% CI = 1.34–2.97) for the no metastasis group, 2.24 (95% CI = 1.45–3.47) for the metastasis group, and 2.00 (95% CI = 1.42–2.82) for the mixed group consisting of studies that included patients at all stages. A high CAR for subjects with metastasis was associated with a numerically higher value for the hazard ratio than for subjects with no metastasis, but this difference was not statistically significant (p for subgroup difference = 0.91). Cancer type subgroups were generated by the number of studies on same cancer if at least two studies on that cancer were available, while the remaining studies were pooled in a subgroup termed “others.” The effect of CAR on OS was significant for all cancer types, and there was no difference between these groups (p = 0.39). All results of subgroup analyses are illustrated in Table 3.

**Figure 4:**
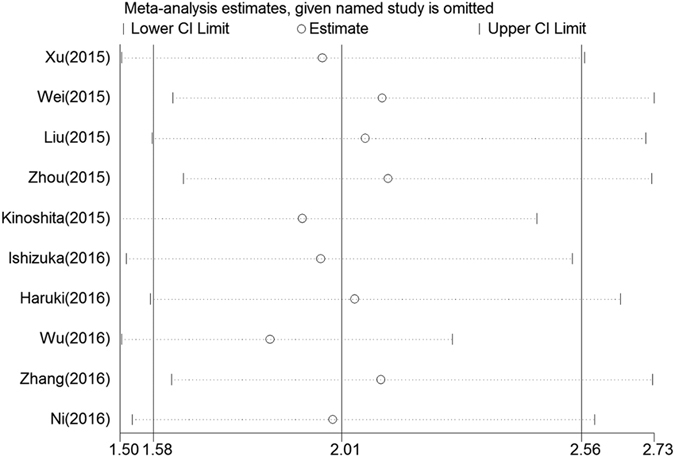
Sensitivity analysis of the relationship between CAR and OS.

**Figure 5:**
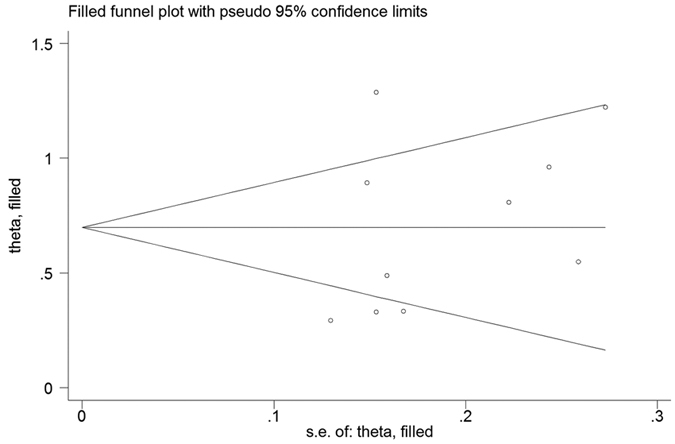
Filled funnel plots for publication bias test of OS.

We conducted multivariate meta-regression analysis to explore the possible source of heterogeneity. The results suggested that cutoff value (p = 0.451), cutoff value selection (p = 0.364), treatment method (p = 0.338), country (p = 0.154), sample size (p = 0.888), stage (p = 0.194) and cancer type (p = 0.682) did not contribute to the heterogeneity (Table 3). A sensitivity analysis was used to determine whether any study could affect the pooled HRs, and the answer was negative (Fig. 4). Begg’s test and Egger’s linear regression test were performed to evaluate the publication bias. Evidence for significant publication bias for OS was not found, as the p value for Begg’s test was 0.283, and the p value for Egger’s test was 0.325. As estimated by the trim-and-fill method, no missing studies were required to make the filled funnel plots symmetrical (Fig. 5).

## Discussion

To the best of our knowledge, no meta-analyses assessing the correlation of CAR with the prognosis and survival of patients with various tumors have been performed. In this study, we combined the outcomes of 4,592 patients from 10 available studies, indicating that a high pretreatment CAR was significantly associated with poor OS HR (2.01, 95% CI: 1.58–2.56, p < 0.001) in different solid cancers, although there was heterogeneity. Subgroup analyses between CAR and OS were performed, and a high CAR was still a negative maker for worse OS when the patients were segregated according to cutoff value, cutoff value selection, treatment method, country, sample size, stage and cancer type.

When stratified by stage, there was a trend for the association of increased CAR with a worse OS to be greater for patients with metastasis than the patients without metastasis. The reason may be that greater tumor burden resulted in more prolonged chronic inflammation. In a NPC study, patients with Stage III-IV disease had a significantly higher pretreatment CRP/Alb ratio than patients with Stage I-II disease. There was no single study affecting the results in our meta-analysis as determined by the sensitivity analysis. Although three methods of testing the publication bias were performed, and the results showed that there was no significant publication bias in our study, the tests may have false negatives due to the small number of studied included in our study. Taken together, the results suggested that some publication bias was likely to be still present and the actual effect sizes could be smaller than we reported.

A critical problem of our meta-analysis was the large range of cutoff values. To address this problem, we analyzed the correlation between the cutoff value and HR for OS. The results showed there was no relationship between the cutoff value and HR for OS. Next, we performed a subgroup analysis based on a lower cutoff value group and a higher cutoff value group. The results showed that there was no difference between these two groups. Another key point was to analyze the selection of the cutoff value. Of the ten included studies, seven studies reported that the cutoff was selected by the receiver operating characteristic (ROC), and the rest 3 studies were based on Cutoff Finder. Furthermore, cutoff selection subgroup analysis showed that elevated CAR was positively related to poor HR both in the ROC group and Cutoff Finder group.

During the initiation of carcinogenesis, the reactive oxygen species (ROS) and reactive intermediates (RNI) released by inflammatory cells induce DNA damage and genomic instability. During this process, cancer cells often over-express proinflammatory mediators, including proteases, cytokines, and chemokines, which activate many inflammatory signaling pathways. The essential role of the inflammatory microenvironment in tumors has been emphasized over the past decades^38^,^39^,^40^. CRP is synthesized by the liver, secreted into the circulation and extensively influenced by proinflammatory cytokines, such as interleukin-6, interleukin-1, tumor necrotic factor-α and transforming growth factor-β^41^. It has been shown to be a sensitive prognosis predictor of colorectal cancer^42^, prostate cancer^43^ and others. However, inflammation and malnutrition may inhibit the production of albumin^44^. The serum albumin level at later stages of tumorigenesis could be significantly decreased by tumor necrotic factor increased permeability of the microvasculature and interleukin -Ib and interleukin-6 induced suppressed albumin synthesis, whereas there was no or slight hypoalbuminemia at the beginning of the disease. Therefore, serum albumin is also good indicator of cancer prognosis^45^,^46^. Thus, we hypothesized that CAR may be a potential biomarker of outcomes for different cancers.

The CAR was first proposed by Fairclough *et al*.^15^ to identify acutely sick patients, and it was later shown to independently predict the mortality of patients with severe sepsis or septic shock^47^, until Kinoshita *et al*.^16^ combined its prognostic value with hepatocellular carcinoma. Previous researchers have demonstrated that GPS, mGPS, NLR and PLR predicted the prognosis of patients with cancer^48^,^49^,^50^. The CAR reflects the ratio of the CRP and albumin levels continuously compared with GPS or mGPS, which may be underestimated or overestimated in some patients. Additionally, CAR showed the highest area under the receiver operating characteristic (AUROC) among these clinical characteristics, such as NLR, CEA, and pathological differentiation in terms of OS^32^.

There were many limitations in this study that need to be carefully considered. First, the number of included studies was limited, and the pooled results may be less powerful. Second, the heterogeneity among these studies was relatively high and could not be eliminated completely. However, a meta-regression analysis was performed, and we did not find that heterogeneity was caused by cutoff value or other factors included in the analysis. The heterogeneity of the study was probably due to other factors, such as different start time to follow-up (four studies reported that the start date was diagnosis, in one study, the start date was the surgery date, and the others study did not report the start date). We could not analyze all the different factors because only summarized data rather than individual patient data could be used. Third, all of the eligible studies were retrospective due to a lack of a relevant prospective studies. Moreover, Zhen Chen’s and Masatsune Shibutani’s studies might contain important parameters for our study. However, we could not include them because of insufficient data. Fourth, publication bias may be present in our meta-analysis because there were no studies with negative results included in this study. Although Egger’s test and the “trim and fill” method were performed to evaluate the publication bias, there may be false negative results due to the limited number of studies. Therefore, whether CAR is a potential prognostic predictor in patients with cancer requires further investigation.

In conclusion, our meta-analysis showed that high CAR was significantly associated with poor OS in patients with cancer. However, further large prospective studies should be conducted to explore the critical role of CAR for survival in cancer patients. If replicated in further large-scale and well-designed studies, our findings will support the clinical use of CAR as a pan-cancer prognostic marker.

## Additional Information

**How to cite this article**: Li, N. *et al*. Prognostic Role of the Pretreatment C-Reactive Protein/Albumin Ratio in Solid Cancers: A Meta-Analysis. *Sci. Rep.* **7**, 41298; doi: 10.1038/srep41298 (2017).

**Publisher's note:** Springer Nature remains neutral with regard to jurisdictional claims in published maps and institutional affiliations.

## Figures and Tables

**Table 1:** Characteristics of the included studies.

Author	Country	Year	Cancer type	Sample size	Study type	Cut-off value of CAR	Range of CAR	Cut-off Selection	Survival analysis	Treatment methods	Follow-up (Median months)	Stage	Age (Range)	NOS score
Kinoshita^16^	Japan	2015	HCC	186	R	0.037	NR	ROC	OS	With-surgery	72	Mixed	43–91	9
Xu^17^	China	2015	ESCC	468	R	0.5	NR	ROC	OS	With-surgery	49.9	I II III	58^Median^	8
Wei^30^	China	2015	ESCC	423	R	0.095	0–7.9	Cutoff Finder	OS	With-surgery	35.7	Mixed	24–88	8
Liu^31^	China	2015	GC	455	R	0.025	NR	ROC	OS	With-surgery	25	I II III	19–86	8
Zhou^29^	China	2015	SCLC	367	R	0.441	NR	Cutoff Finder	OS	No-surgery	29.4	Mixed	23–82	8
Ishizuka^32^	Japan	2016	CRC	627	R	0.038	NR	ROC	OS	With-surgery	29.97	Mixed	NR	8
Haruki^34^	Japan	2016	PC	113	R	0.03	NR	ROC	OS/DFS	With-surgery	NR	Mixed	27–85	8
Wu^35^	China	2016	PC	233	R	0.54	0.002–6.728	Cutoff Finder	OS	No-surgery	NR	Mixed	26–85	8
Zhang^36^	China	2016	NPC	1572	R	0.05	0.002–4.594	ROC	OS/DFS	No-surgery	50	Mixed	14–78	7
Ni^33^	China	2016	CRC	148	R	0.6712	NR	ROC	OS	No-surgery	12	IV	20–74	7

HCC: hepatocellular carcinoma; ESCC: esophageal squamous cell carcinoma; GC: gastric cancer; SCLC: small cell lung cancer; CRC: colorectal cancer; PC: pancreaticcancer; NPC: nasopharyngeal carcinoma; R: retrospective; OS: overall survival; ROC: the receiver operating characteristic; DFS: disease-free survival; NOS: the newcastle-ottawa quality assessment scale; NR: not reported.

**Table 2:** Assessment of Study Quality.

Author	Selection				Comparability			Outcome		Score
	Item 1	Item 2	Item 3	Item 4	Item 5	Item 6	Item 7	Item 8	Item 9	
Kinoshita^16^	*	*	*	*	*	*	*	*	*	9
Xu^17^	*	*	*	*	*	*	*	*	-	8
Wei^30^	*	*	*	*	*	*	*	*	-	8
Liu^31^	*	*	*	*	-	*	*	*	*	8
Zhou^29^	*	*	*	*	*	*	*	*	-	8
Ishizuka^32^	*	*	*	*	*	*	*	*	-	8
Haruki^34^	*	*	*	*	-	*	*	*	*	8
Wu^35^	*	*	*	*	*	*	*	*	-	8
Zhang^36^	*	*	*	*	-	*	*	*	-	7
Ni^33^	*	*	*	*	*	*	*	-	-	7

-: zero point, *: one point, Item 1: representativeness of the exposed cohort; Item 2: selection of the non exposed cohort; Item 3: ascertainment of exposure; Item 4: demonstration that outcome of interest was not present at start of study; Item 5: comparability of cohorts on the basis of the design (study controls for the most important factor, including infection or other inflammatory conditions); Item 6: comparability of cohorts on the basis of the design (study controls for any additional factor, including age, gender and stage); Item 7: assessment of outcome; Item 8: follow-up long enough for outcomes to occur; Item 9: adequacy of follow-up of cohorts.

**Table 3:** Results of subgroup meta-analysis and meta-regression analysis.

Subgroup	No. of studies	No. of patients	HR(95%CI)	Heterogeneity		Meta-regression P value	Subgroup difference P value
			Random-effects model	Ph	I^2^(%)		
**Cutoff Value**						0.451	0.40
Lower cutoff	6	3376	1.81(1.40–2.34)	0.03	61		
Higher cutoff	4	1216	2.26(1.44–3.56)	<0.001	92		
**Cutoff Value Selection**						0.364	0.80
ROC	7	3569	2.05(1.64–2.57)	0.03	56		
Cutoff finder	3	1023	1.89(1.01–3.51)	< 0.001	93		
**Treatment**						0.338	0.92
With-surgery	6	2272	2.03(1.56–2.64)	0.01	65		
No-Surgery	4	2320	1.97(1.20–3.23)	< 0.001	90		
**Country**						0.154	0.25
China	7	3666	1.87(1.40–2.50)	< 0.001	84		
Japan	3	926	2.47(1.70–3.59)	0.19	40		
**Sample size**						0.888	0.03
≥300	6	3912	1.69(1.35–2.12)	0.01	67		
<300	4	680	2.69(1.90–3.81)	0.05	61		
**Stage**						0.194	0.91
Mixed	7	3521	2.00(1.42–2.82)	< 0.001	84		
No-Metastasis	2	923	2.00(1.34–2.97)	0.06	71		
Metastasis	1	148	2.24(1.45–3.47)				
**Cancer Type**						0.682	0.39
ESCC	2	891	1.84(1.06–3.19)	0.009	85		
PC	2	346	2.58(1.25–5.31)	0.01	83		
CRC	2	775	2.40(1.74–3.32)	0.64	0		
Others	4	2580	1.68(1.23–2.29)	0.02	70		

ESCC: esophageal squamous cell carcinoma; PC: pancreatic cancer; CRC: colorectal cancer; HR: hazard ratio; 95%CI: 95% confidence interval; Ph: p-value of Q test for heterogeneity test.

## References

[b1] Vineis P. & Wild C. P. Global cancer patterns: causes and prevention. Lancet 383, 549–57 (2014). 24351322 10.1016/S0140-6736(13)62224-2

[b2] Felder M. . MUC16 (CA125): tumor biomarker to cancer therapy, a work in progress. Molecular cancer 13, 129, doi: 10.1186/1476-4598-13-129 (2014). 24886523 PMC4046138

[b3] Vickers A. J., Thompson I. M., Klein E., Carroll P. R. & Scardino P. T. A commentary on PSA velocity and doubling time for clinical decisions in prostate cancer. Urology 83, 592–6 (2014). 24581521 10.1016/j.urology.2013.09.075

[b4] Grivennikov S. I., Greten F. R. & Karin M. Immunity, inflammation, and cancer. Cell 140, 883–99 (2010). 20303878 10.1016/j.cell.2010.01.025 PMC2866629

[b5] Balkwill F. & Mantovani A. Inflammation and cancer: back to Virchow? Lancet 357, 539–45 (2001). 11229684 10.1016/S0140-6736(00)04046-0

[b6] Hussain S. P. & Harris C. C. Inflammation and cancer: an ancient link with novel potentials. Int J Cancer 121, 2373–80 (2007). 17893866 10.1002/ijc.23173

[b7] Shalapour S. & Karin M. Immunity, inflammation, and cancer: an eternal fight between good and evil. J Clin Invest 125, 3347–55 (2015). 26325032 10.1172/JCI80007 PMC4588298

[b8] Salman T. . Prognostic Value of the Pretreatment Neutrophil-to-Lymphocyte Ratio and Platelet-to-Lymphocyte Ratio for Patients with Neuroendocrine Tumors: An Izmir Oncology Group Study. Chemotherapy 61, 281–6 (2016). 27070366 10.1159/000445045

[b9] You J. . Preoperative platelet to lymphocyte ratio is a valuable prognostic biomarker in patients with colorectal cancer. Oncotarget (2016). 10.18632/oncotarget.8334 PMC5041922 27027440

[b10] Nakamura Y. . Neutrophil/lymphocyte ratio has a prognostic value for patients with terminal cancer. World J Surg Oncol 14, 148 (2016). 27184053 10.1186/s12957-016-0904-7 PMC4867538

[b11] Omae K., Kondo, T. & Tanabe K. High preoperative C-reactive protein values predict poor survival in patients on chronic hemodialysis undergoing nephrectomy for renal cancer. Urol Oncol 33, 67.e9-13 (2015). 10.1016/j.urolonc.2014.07.004 25130069

[b12] Saito K. & Kihara K. Role of C-reactive protein in urological cancers: a useful biomarker for predicting outcomes. Int J Urol 20, 161–71 (2013). 22897628 10.1111/j.1442-2042.2012.03121.x

[b13] Omichi C., Nakamura K., Haraga J., Masuyama H. & Hiramatsu Y. Glasgow prognostic score is an independent marker for poor prognosis with all cases of epithelial ovarian cancer. Cancer Med (2016). 10.1002/cam4.681 PMC4924365 26929186

[b14] Zhang X. . Modified glasgow prognostic score as a prognostic factor in gastriccancer patients: a systematic review and meta-analysis. Int J Clin Exp Med 8, 15222–9 (2015). 26629007 PMC4658896

[b15] Fairclough E., Cairns E., Hamilton J. & Kelly C. Evaluation of a modified early warning system for acute medical admissions and comparison with C-reactive protein/albumin ratio as a predictor of patient outcome. Clin Med (Lond.) 9, 30–3 (2009). 19271597 10.7861/clinmedicine.9-1-30 PMC5922628

[b16] Kinoshita A. . The C-reactive protein/albumin ratio, a novel inflammation-based prognostic score, predicts outcomes in patients with hepatocellular carcinoma. Ann Surg Oncol 22, 803–10 (2015). 25190127 10.1245/s10434-014-4048-0

[b17] Xu X. L., Yu H. Q., Hu W., Song Q. & Mao W. M. A Novel Inflammation-Based Prognostic Score, the C-Reactive Protein/Albumin Ratio Predicts the Prognosis of Patients with Operable Esophageal Squamous Cell Carcinoma. PLoS One 10, e0138657 (2015). 26390126 10.1371/journal.pone.0138657 PMC4577080

[b18] Moher D., Liberati A., Tetzlaff J., Altman D. G. & Group P. Preferred reporting items for systematic reviews and meta-analyses: the PRISMA statement. Bmj 339, b2535, doi: 10.1136/bmj.b2535 (2009). 19622551 PMC2714657

[b19] Stang A. Critical evaluation of the Newcastle-Ottawa scale for the assessment of the quality of nonrandomized studies in meta-analyses. European journal of epidemiology 25, 603–605, doi: 10.1007/s10654-010-9491-z (2010). 20652370

[b20] Zhang J. . Pretreatment Lymphocyte Monocyte Ratio Predicts Long-Term Outcomes in Patients with Digestive System Tumor: A Meta-Analysis. Gastroenterology research and practice 2016, 9801063, doi: 10.1155/2016/9801063 (2016). 27594882 PMC4993921

[b21] Tierney J. F., Stewart L. A., Ghersi D., Burdett S. & Sydes M. R. Practical methods for incorporating summary time-to-event data into meta-analysis. Trials 8, 16 (2007). 17555582 10.1186/1745-6215-8-16 PMC1920534

[b22] DerSimonian R. & Laird N. Meta-analysis in clinical trials. Control Clin Trials 7, 177–88 (1986). 3802833 10.1016/0197-2456(86)90046-2

[b23] Mantel N. & Haenszel W. Statistical aspects of the analysis of data from retrospective studies of disease. J Natl Cancer Inst 22, 719–48 (1959). 13655060

[b24] Begg C. B. & Mazumdar M. Operating characteristics of a rank correlation test for publication bias. Biometrics 50, 1088–1101 (1994). 7786990

[b25] Egger M., Davey Smith G., Schneider M. & Minder C. Bias in meta-analysis detected by a simple, graphical test. Bmj 315, 629–634 (1997). 9310563 10.1136/bmj.315.7109.629 PMC2127453

[b26] Duval S. & Tweedie R. Trim and fill: A simple funnel-plot-based method of testing and adjusting for publication bias in meta-analysis. Biometrics 56, 455–463 (2000). 10877304 10.1111/j.0006-341x.2000.00455.x

[b27] Shibutani M. . Prognostic Significance of the Preoperative Ratio of C-Reactive Protein to Albumin in Patients with Colorectal Cancer. Anticancer Res 36, 995–1001 (2016). 26976989

[b28] Chen Z. . Prognostic significance of preoperative C-reactive protein: albumin ratio in patients with clear cell renal cell carcinoma. Int J Clin Exp Pathol 8, 14893–900 (2015). 26823819 PMC4713605

[b29] Zhou T. . Ratio of C-Reactive Protein/Albumin is An Inflammatory Prognostic Score for Predicting Overall Survival of Patients with Small-cell Lung Cancer. Scientific Reports 5, 10481 (2015). 10.1038/srep10481 PMC4471724 26084991

[b30] Wei X. L. . A novel inflammation-based prognostic score in esophageal squamous cell carcinoma: the C-reactive protein/albumin ratio. BMC Cancer 15, 350 (2015). 25934640 10.1186/s12885-015-1379-6 PMC4423167

[b31] Liu X. . Preoperative C-Reactive Protein/Albumin Ratio Predicts Prognosis of Patients after Curative Resection for Gastric Cancer. Transl Oncol 8, 339–45 (2015). 26310380 10.1016/j.tranon.2015.06.006 PMC4562973

[b32] Ishizuka M. . Clinical Significance of the C-Reactive Protein to Albumin Ratio for Survival After Surgery for Colorectal Cancer. Ann Surg Oncol 23, 900–7 (2016). 26530445 10.1245/s10434-015-4948-7

[b33] Ni X. F. . C-reactive protein/albumin ratio as a predictor of survival of metastatic colorectal cancer patients receiving chemotherapy. International journal of clinical and experimental pathology 9, 5525–5534 (2016).

[b34] Haruki K. . The C-reactive Protein to Albumin Ratio Predicts Long-Term Outcomes in Patients with Pancreatic Cancer After Pancreatic Resection. World J Surg (2016). 10.1007/s00268-016-3491-4 26956901

[b35] Wu M., Guo J., Guo L. & Zuo Q. The C-reactive protein/albumin ratio predicts overall survival of patients with advanced pancreatic cancer. Tumor Biology (2016). 10.1007/s13277-016-5122-y PMC5080377 27344157

[b36] Zhang Y. . Exploration and Validation of C-Reactive Protein/Albumin Ratio as a Novel Inflammation-Based Prognostic Marker in Nasopharyngeal Carcinoma. Journal of Cancer 7, 1406–1412, doi: 10.7150/jca.15401 (2016) 27471556 PMC4964124

[b37] Budczies J. . Cutoff Finder: a comprehensive and straightforward Web application enabling rapid biomarker cutoff optimization. PloS one 7, e51862, doi: 10.1371/journal.pone.0051862 (2012). 23251644 PMC3522617

[b38] Fernandes J. V. . The role of the mediators of inflammation in cancer development. Pathol Oncol Res 21, 527–34 (2015). 25740073 10.1007/s12253-015-9913-z

[b39] Colotta F., Allavena P., Sica A., Garlanda C. & Mantovani A. Cancer-related inflammation, the seventh hallmark of cancer: links to genetic instability. Carcinogenesis 30, 1073–81 (2009). 19468060 10.1093/carcin/bgp127

[b40] Candido J. & Hagemann T. Cancer-related inflammation. J Clin Immunol 33 Suppl 1, S79–84 (2013). 23225204 10.1007/s10875-012-9847-0

[b41] Morris-Stiff G., Gomez D. & Prasad K. R. C-reactive protein in liver cancer surgery. Eur J Surg Oncol 34, 727–9 (2008). 18356004 10.1016/j.ejso.2008.01.016

[b42] Nimptsch K. . Association of CRP genetic variants with blood concentrations of C-reactive protein and colorectal cancer risk. Int J Cancer 136, 1181–92 (2015). 25043606 10.1002/ijc.29086 PMC6284796

[b43] Xu L. . Serum C-reactive protein acted as a prognostic biomarker for overall survival in metastatic prostate cancer patients. Tumour Biol 36, 669–73 (2015). 25286759 10.1007/s13277-014-2670-x

[b44] Yeun J. Y. & Kaysen G. A. Factors influencing serum albumin in dialysis patients. Am J Kidney Dis 32, S118–25 (1998). 10.1016/s0272-6386(98)70174-x 9892378

[b45] Barber M. D., Ross J. A. & Fearon K. C. Changes in nutritional, functional, and inflammatory markers in advanced pancreatic cancer. Nutr Cancer 35, 106–10 (1999). 10693162 10.1207/S15327914NC352_2

[b46] McMillan D. C. . Albumin concentrations are primarily determined by the body cell mass and the systemic inflammatory response in cancer patients with weight loss. Nutr Cancer 39, 210–3 (2001). 11759282 10.1207/S15327914nc392_8

[b47] Kim M. H. . The C-Reactive Protein/Albumin Ratio as an Independent Predictor of Mortality in Patients with Severe Sepsis or Septic Shock Treated with Early Goal-Directed Therapy. PLoS One 10, e0132109 (2015). 26158725 10.1371/journal.pone.0132109 PMC4497596

[b48] Yamada S. . Clinical Implication of Inflammation-Based Prognostic Score in Pancreatic Cancer: Glasgow Prognostic Score Is the Most Reliable Parameter. Medicine (Baltimore) 95, e3582 (2016). 27149487 10.1097/MD.0000000000003582 PMC4863804

[b49] Zhou X. . Prognostic value of PLR in various cancers: a meta-analysis. PLoS One 9, e101119 (2014). 24968121 10.1371/journal.pone.0101119 PMC4072728

[b50] Cho I. R. . Pre-treatment neutrophil to lymphocyte ratio as a prognostic marker to predict chemotherapeutic response and survival outcomes in metastatic advanced gastric cancer. Gastric Cancer 17, 703–10 (2014). 24442663 10.1007/s10120-013-0330-2

